# The World's Columbian Dental Congress

**Published:** 1894-03

**Authors:** George Cunningham


ARTICLE IV.

### THE WORLD'S COLUMBIAN DENTAL CONGRESS.

BY GEORGE CUNNINGHAM.

Some members of the Odontological Society of Great Britain may be interested in the following comments on three items which, we have reason to believe, figured in the discussion of the World's Congress Auxiliary circular, and led to the rescinding of the resolution of a previous meeting at which delegates had been appointed to the Congress.

Firstly, as to what we may briefly characterize as the erroneous statements of America being the birthplace and home of scientific dentistry; I made inquiries in many directions, but found that very few had ever seen the circular in question, and not a single one of those who had perused this wretched and unfortunate publication, but condemned the objectionable passages in the strongest terms.

Secondly, apprehension was expressed as to the two public meetings which were arranged to be held in conformity with the general rules appertaining to all the congresses. It is true the public were admitted to the gallery during the general meetings, but were absolutely excluded from the sectional meetings and the clinics. As a matter of fact, the attendance in the gallery was of the very scantiest, and consisted mainly of the relations and friends of dentists and a few dental students. Indeed, I scarcely think that the Dental Executive quite carried out the intentions of the Congress Auxiliary Executive. I certainly anticipated that the two popular evening meetings would have been devoted to the treatment and discussion of some features of dentistry from the standpoint of public interest, such as a lantern lecture on dental hygiene, or a discussion under the superintendence of the committee on the care of the teeth of the poor. As a matter of fact, neither of the evening general meetings, though well attended by the members of the Congress, were in any sense popular, but were devoted to lantern exhibits illustrating purely professional subjects, which were treated in a way worthy of the Odontological Society itself.

Thirdly, with regard to the Women's Branch, "The officials of the World's Columbian Dental Congress have shown the most favorable disposition towards women dentists, but the women themselves have been slow to respond," as mentioned by the recording secretary in her report to the meeting of the Women's Dental Association of the United States, held during the progress of the Congress. As far as I could gather, about twenty to twenty-five women dentists attended the Congress, all of whom were members of recognized dental societies, with the exception of three foreign representatives. They contributed some papers of no uncommon interest in several sections, including one on "Mercuric Chloride as a Germicide," which showed a familiarity with the operations of bacteriological research as yet possessed by few dentists.

It is curious how such an episode as this unfortunate circular may warp one's common sense, culminating in attributing ulterior motives to even commonplace incidents. It seems that the official letter from the Odontological Society of Great Britain was returned from America unopened. Some of my friends, more or less touched with Americaphobia, seemed to think that this was a part of the wily tactics of the Congress Executive, and asked me if I could give any explanation of the fact. I confidently expressed the opinion that it was a matter entirely connected with the postal service. I therefore naturally took an early opportunity of ascertaining the true history of the returned letter. The letter was addressed to the President of the World's Columbian Dental Congress at

Chicago. No such person was known or could be found by the postal authorities; and, therefore, after a time, as is usual in such cases, the letter was returned to the sender. It was then re-despatched to America, but this time addressed to the president personally, at his home address in Boston, where, of course, it duly reached him, but only a few days before the beginning of the Congress. I know that it was a matter of great regret to him; firstly, that the letter had miscarried, and secondly, that in the midst of pressure of work he had not had the opportunity of sending an official reply before the Congress.

Now to return to our "moutons," no disrespect meant, most of our friends were busily engaged on executive work or in connection with the meetings of the National Association of Dental Faculties and the National Association of Dental Examiners, which were being held antecedent to the Congress. These two bodies are doing extremely good and useful work, especially in standardizing the dental education in the different States. Their existence is a practical recognition that leaving the control of higher professional education to bodies with naturally diversified State laws is bad, and that such matters are of federal, or as we should say, imperial concern. It is indeed a matter on which we may congratulate ourselves that there is no necessity for analagous bodies in this country, owing to the absolute identity of the dental laws in the several parts of the United Kingdom, and the practical uniformity of the dental curriculum in all our dental educational institutions. It might, however, be worth while to consider the advisability of an occasional conference of the executive or delegates from our different dental schools to discuss how far, and in what respects, the present curriculum, framed many years ago, might be improved.

The following quotation from the proceedings of the latter of these two bodies as to the number of students during the session of 1892-93 is a striking contrast to the number of students recently attending our own schools, which is so small as to give rise to the very serious question whether the increase in the number of students is proportionate to the requirements of the community. "Session 1892-93, number of students in recognized schools: Freshmen, 1,429; Juniors, 927; Seniors, 433; Graduates, 320; Post-graduates, 44 (one school not reported). Total, 3,153. In unrecognized schools the number of students were: Freshmen, 111; Juniors, 54; Seniors, 22; Graduates, 20." Nor does this statement quite exhaust the actual number of students, for there are probably many studying with private practitioners, who might fairly be ranked as students, especially for the purposes of comparison, with the number studying in this country; since it must be remembered that in the United States it is still possible for a student to become a legally qualified practitioner without necessarily passing through a dental college. In this respect British legislation is much in advance of even the State legislation in the United States, since it is impossible for any student of dentistry to become a legally qualified practitioner without (1) passing a preliminary examination in arts similar to that required of medical students; (2) four years spent in professional education, which includes three years practical instruction in mechanical dentistry under a registered dental practitioner, or under the direction of the superintendent of the mechanical department of a recognized dental hospital, and two years devoted to lectures and practical work, attendance being obligatory both at a medical and a dental school, before he can enter for the qualifying examination which is always conducted by one of the licensing corporations having no connection with the teaching bodies.

We had plenty of opportunity of comparing notes on this and other matters with the various practitioners who were now beginning to throng the reception-rooms of the Columbian Dental Club. Our acquaintance with the American practitioners was simply increasing by leaps and bounds. The weather was hot enough to make us revel in the cooling lager, or enjoy the soothing effects of a cigar, seated in the shade, surrounded by good fellows who betrayed not the slightest trace of animosity, even in discussing our international differences. Yes, the Columbian Dental Club was indeed a great institution. It was very conveniently and centrally situated, and became the general headquarters of every dentist who visited Chicago, and at times, even its large rooms were thronged to almost overcrowding. From early morn, one might almost say, to early morn, the Chicago dentists thus kept open house to one and all. It was not a palace like the Union League Club or the Chicago Athletic; it was not luxuriously furnished, but it was spacious, comfortably equipped, and served its temporary purpose so well that many will regret that it could not become a permanent institution. Not the least best remembered feature of the Congress to most visitors will be the pleasant days and nights spent in or around and about the Columbian Dental Club, nor will they readily forget the valuable and innumerable services of the omnipresent managerial, Brophy, nor those of his colored henchman "the Commodore."

You, Mr. Editor, have well remarked that "there is the social side of a meeting, under its soothing influence, begotten largely of better knowledge, angularities get rounded off, annoyances allayed, and mutual appreciation heightened." The Club contributed enormously to the development of the social side of the Congress, far more, indeed, that any amount of separate receptions and more or less formal entertainments could have done. It is to be hoped, therefore, that it is but the forerunner of a similar institution at the next dental congress, wherever that may be. Paris, 1900 ? Well, perhaps, but why not before ? Many a time during this professional holiday, both Americans and foreigners have spontaneously remarked that the next dental congress should be in England; and why not ? I frankly admit that I discouraged any such anticipations, mainly because so many British dentists regard an independent dental congress as inimical to the dental section of the International Medical Congress. Is not our policy in this regard rather contradictory, not to say inconsistent; since it is a well known fact that as a profession we have discouraged, nay prevented, the formation of a dental section of the British Medical Association, because that would interfere with the prosperity of our independent Dental Association and its independent Annual General Meeting? As a lover of all good things, I am an advocate for both, and see no reason why we sbould not hold, from time to time, independent international congresses, such as are now held by many sections and specialties of the medical profession, while according our fullest support to all international congresses where dentistry is concerned, or is capable of playing an important part, such as the International Medical Congress or that of Hygiene and Demography.

I remember urging upon a friend, as a reason why he should give the Congress his fullest support by accepting an honorary officership, that the membership was to be confined to legally qualified and reputable dentists (as defined in the code of ethics in the American and Southern Dental Association) residing in the United States, and such dentists residing in foreign countries who were certificated eligible by the honorary officers of their respective countries, to which his reply was—"Oh, yes; that all looks very well on paper, but they won't carry it out." While seated at the Club one night, I received a message from the Secretary-General requesting me, in the absence of either of the foreign secretaries, to interview a gentleman who could not talk English, but wanted to make use of the Club and enroll himself as a member of the Congress. He described himself as a dentist practising in Belgium, and on my asking him if he had the necessary certificate as to his eligibility from the honorary officers in Belgium, he said that he had not, and that he had never heard of the regulation, as he had been in Chicago since the opening of the Exhibition. On his learning that none of the officers representing Belgium were present, he very kindly volunteered to give an address on behalf of Belgium at the opening ceremony. I naturally inquired how it was that he, a dentist, had been able to be so long in Chicago, when he informed me that he had charge of an exhibit which was creating great interest at the World's Fair, and exhibited quite a bundle of cards of many eminent American practitioners, who he said had expressed great admiration of his exhibit. As I knew that several of the gentlemen, whose cards he produced, did not talk French, and he did not talk English, I did not attach much importance to that. Further inquiries elicited the fact that he also had charge of a perfumery stall, that he belonged to a family of seventeen, all dentists, and that he was the author of a "Guide Pratique d'Hygiene and d'Art Dentaires," a copy of which he kindly presented to me. I need scarcely say that his name and address was prominent on the title page, followed by the following astounding catalogue of his qualifications :—"Dentiste agree de differentes ecoles de la province de Liege, Spa, Ostende, etc ; Plusieurs fois brevete, membre de jury hors concurs; Medailles d'or et diplomes d'honneur aux Expositions d'Amsterdam, 1883; Londres, 1884; Ostende, 1887; Nice et Tunis, 1888; Paris, 1889; et Liege, 1890; Recompense par le Gouvernement Belge et la ville de Liege." This tissue of self-fabricated honors was followed by the announcement that the manuscript of this work had been awarded a prize in different exhibitions. A glance at the preface showed that his previous pamphlet on the same subject had met with a reception at the hands of the public which surpassed all his fondest hopes—40,000 copies had been disposed of (enleves) in a few months—and that he made no pretence to science, but desired simply to let the public profit by the fruits of his own experience and practical notions, which study had enabled him to acquire from the most authoritative sources. I

need scarcely say that the application of this philanthropist was promptly rejected. Notwithstanding this rebuff he managed to secure, in some way or other, about the second or third day of the Congress, a ticket of membership as a foreign member. He was, however, soon after recognized; his ticket of membership was cancelled, and his magic button promptly confiscated.

It is therefore worth noting that the executive of the World's Columbian Dental Congress honestly endeavored to carry out the ethical restrictions it had imposed as to membership; indeed, so far as I know, it is the first time on record in which any congress, either medical or dental, has endeavored to rigorously exact any such ethical qualification as to membership. Of course it is almost impossible, in the pressure of business, especially during the earlier days, to absolutely prevent some fraudulent registrations. During the course of the Congress it transpired that some six persons, four from the United States and two from abroad, who had succeeded in registering as members, were afterwards found to be ineligible, their membership was therefore annulled and their subscriptions returned. Before the final authoritative list of members is published, it is the intention of the Executive Committee to further overhaul the list, and return the fees of those whose membership may be canceled. There can be no doubt that many a quack and advertising practitioner, especially from foreign countries, is admitted without cavil to these large congresses; and we would especially recommend the adoption of this practice of placing the responsibility of foreign membership on the honorary officers of their respective countries, as otherwise angels are sure to be entertained unawares.

The conjoint dental societies of Chicago gave the Congress a good send-off in the shape of a reception at Kinsley's (the Whitehall rooms of Chicago). It was indeed a splendid success, mainly owing to the number and the attractions of the ladies present, though, perhaps, I say it as should not, since I learn from a comment iu the *Dental Tribune* "that England's representatives (*incog.*) did much to remove the formal frigidity of the atmosphere by starting up a few dances."

About this time the Executive Committee requested me to undertake the duty of replying on behalf of Great Britain at the opening ceremony. Having already, in conjunction with my colleagues, declined my appointment as honorary secretary, I felt a considerable delicacy in doing so, until I was assured by the other representatives of Great Britain present that some one ought to reply on behalf of our country and that I should do it.

The World's Columbian Dental Congress was formally convened at the Hall of Washington, in the Memorial Art Palace, Chicago, Monday, August 14, 1893. The proceedings of that day were of the most auspicious character, and from that moment forward there was no doubt as to the success of the Congress; the attendance was magnificient and truly international from the unusual number of representatives from foreign lands. President Bonney, of the World's Congress Auxiliary, delivered an address, which was short and to the point, welcoming us to Chicago and introducing Dr. Walker, President of the Executive Committee, who in a short, pithy, pointed speech introduced the various officers of the Congress. It must have been rather a trying introduction, I thought, for some of them, but it is a useful although a somewhat uncomfortable kind of ceremony.

The Secretary-General, Dr. Harlan, then read the resolutions creating the Congress. For obvious reasons, although it was no speech, the speaker was hailed with a perfect storm of applause, an eloquent tribute of appreciation of an infinity of hard work. In the absence of the Hon. John Temple Graves, who was to have delivered an address entitled "The Needs of Dentistry from the Public Standpoint," the Congress found an eloquent and able substitute in the person of Dr. Crawford, of Nashville, who delivered certainly a stirring address of welcome. Both the matter and the delivery, a Britisher would say, were essentially American. He would not be quite right, but it is the only one of the types of American oratory. We all try to rise to great occasions and usually fail—there was no kind of failure about this speech. But it must be admitted that to the timid foreign representative there is something truly appalling in stentorian tones, full of earnest conviction, "that quite seventy million hearts to-day are expressing through the orator their universal welcome to the dentists of the world." It is a sublime thought, of course, but how difficult to live up to it. Then when he asked us, gentlemen from the foreign countries, "to grasp his hand in imagination and feel the pulsation of the great heart of the American people, being in unison at this very moment," I felt almost afraid, for alas! I had no prepared speech committed to memory, and the orator's eloquence made me all the more conscious of my own inadequacy.

The inaugural address of the President of the Congress, as may be gathered from the abstract already published in this journal, was worthy of the great occasion; but he, perhaps, most showed his pre-eminent qualities for the office in the graceful speeches with which he introduced the various foreign representatives. The first on the list was Great Britain, and indeed it was a moment worth living to listen to the President's kindly remarks and the astounding reception with which they were indorsed by that vast audience. As an actor in that, to me at least, ever memorable scene, I do not mind admitting that I was almost overcome. I never anticipated that under the circumstances our country's name could have met with such a reception—honestly, I really seemed to feel the pulsation of the great heart of at any rate the American dentists, and I endeavored to rise to the occasion.—*Extracts from London Dental Record.*

